# Detecting and preventing child maltreatment in primary care and PHNs’ role—a cross-sectional study

**DOI:** 10.1186/s12875-024-02445-x

**Published:** 2024-06-15

**Authors:** Astrid Durdei Midtsund, Lena Henriksen, Mirjam Lukasse, Lisbeth Valla

**Affiliations:** 1 Department of Nursing and Health Promotion, Oslo Metropolitan University, Oslo, Norway; 2 Oslo Municipality, Grünerløkka Child and Family Health Clinic, Oslo, Norway; 3 Division of General Gynaecology and Obstetrics, Oslo University Hospital, Oslo, Norway; 4 Department of Nursing and Social Sciences, Institute of Nursing and Health Sciences, University of South-Eastern Norway, Kongsberg, Norway; 5 Regional Centre for Child and Adolescent Mental Health, Eastern and Southern Norway (RBUP), Oslo, Norway

**Keywords:** Primary Care, Child and Family care, Public Health Nurses, Nursing Care, Child Maltreatment, Child Abuse, Interdisciplinary collaboration, Guidelines

## Abstract

**Background:**

Child maltreatment is a global problem that puts children at risk of mental illness, substance abuse, and premature death. Interdisciplinary collaboration is important in preventing and detecting child maltreatment. In Norway, children undergo universal preventive health assessments and receive complimentary follow-up care from specialized public health nurses in child and family health clinics. These nurses conduct regular check-ups and home visits to monitor children for signs of maltreatment.

**Objective:**

The objective of this study is to describe how public health nurses at child and family health clinics follow the National Clinical Guidelines to prevent and detect child maltreatment, with a particular focus on clinical procedures and interdisciplinary collaboration. Furthermore, we aim to determine factors that are associated with identification of child maltreatment.

**Design:**

A cross-sectional online survey was conducted among public health nurses working in primary care between October 24th and December 31st, 2022. Public health nurses who worked with children aged 0–5 years and had consultations with families were eligible to participate, resulting in 554 responses. The study employed descriptive analysis, including frequency, percentage and mean, as well as a two-step logistic regression analysis. The study was approved by the relevant authority, and informed consent was obtained through questionnaire completion.

**Results:**

The public health nurses in this study displayed strong adherence to the guidelines and utilized various comprehensive assessment procedures to monitor child well-being, growth, and development. However, there was limited and infrequent collaboration with other professionals, such as child protection services, general practitioners, and hospitals. Most public health nurses reported occasional suspicion of child maltreatment, with age and years of experience in child and family clinics influencing these suspicions. Older public health nurses were more likely to suspect physical violence, while those with less than two years of experience reported less experience in suspecting maltreatment. Additional education increased the probability of suspecting sexual violence.

**Conclusions:**

This study provides insights into the practices of public health nurses in Norway regarding the detection and prevention of child maltreatment in child and family clinics. While adherence to guidelines is strong, suspicion of maltreatment is relatively rare. Collaboration across agencies is crucial in addressing child maltreatment. Age and experience may influence the detection of maltreatment. Improved collaboration, targeted guidelines, and ongoing professional development are needed to enhance child protection.

**Supplementary Information:**

The online version contains supplementary material available at 10.1186/s12875-024-02445-x.

## Introduction

Child maltreatment is a significant global problem with severe consequences for children, families, and society [1–8]. Large prospective studies have repeatedly demonstrated that children exposed to child maltreatment have a significantly increased risk of developing mental illness, cognitive impairment, substance abuse, criminal behavior, impaired physical health, and premature death compared to non-maltreated children [8–12]. The World Health Organization (WHO) defines child maltreatment as ‘*all forms of physical and/or emotional ill-treatment, sexual abuse, neglect or negligent treatment or commercial or other exploitation, resulting in actual or potential harm to the child’s health, survival, development or dignity in the context of a relationship of responsibility, trust or power* [1, 13].

The prevalence of child maltreatment varies across studies. Review of a series of meta‐analyses reported an overall estimated prevalence for self-reported studies of 13% for sexual violence, 23% for physical violence, 26% emotional violence and 24% neglect [13]. Child maltreatment is a significant concern in Europe, including Norway [14, 15]. The frequency of various forms of child maltreatment changes according to a child's age. Specifically, sexual violence exhibits higher incidence rates during adolescence, whereas physical violence presents a heightened risk for infants, potentially leading to severe consequences [16]. Early experiences influence how the brain develops and grow [6, 17, 18], and a young child's environment and relationships can either encourage or hinder the healthy development of their brain and related physical and psychological processes. Consequently, early intervention is crucial to prevent negative developmental paths and support positive neurodevelopmental outcomes.

The WHO Regional Office for Europe has been advocating for a shift of focus from a protection-centered approach to child maltreatment to one of prevention [14]. The European report on preventing child maltreatment outline supporting early childhood development, including prenatal development and prevention of maltreatment and adversity, with special attention to children aged 0–3 years [14]. WHO have developed guidelines to provide the evidence base recommendations on parenting interventions to reduce child maltreatment and enhance parent–child relationships [19]. Early interventions, such as home visits and parental support, have shown to be effective in preventing child maltreatment [20, 21]. Further, interdisciplinary collaboration is highlighted as essential in preventive efforts to address the multifaceted nature of child maltreatment [10, 22–25].

Several studies highlight that interdisciplinary collaboration is crucial for obtaining high-quality medical information in detecting child maltreatment, and effective communication and information sharing are considered key factors for successful collaboration [24, 25]. Nevertheless, there is a well-documented deficiency or inadequacy in collaboration and communication among services in the field of child maltreatment [24, 26, 27].

In Norway children receive universal preventive health assessments and complimentary follow-up care provided by specialized public health nurses (PHNs). Child and family health clinics (CFHC) provide a standardized program with regular check-ups and the Health and Care Services Act mandates the inclusion of healthcare professionals, including PHNs, doctors, and physiotherapists, within the staffing requirements. Additionally, midwives are obligated to deliver both antenatal and postnatal care. PHNs are the primary caregivers at CFHCs and like many other countries, Norway has established national clinical guidelines (further referred to 'the guidelines') for primary healthcare personnel to prevent and detect child maltreatment [28].

These guidelines recommend a minimum of 14 check-ups at CFHC from birth to school age with most of them taking place in the first year [29]. The guidelines highlight the importance of consistently monitoring children, remaining vigilant for potential signs of maltreatment and suggest use of various communication tools (see Table 1 for clarification) to reflect and discuss child maltreatment proactively and preventively. The content of the guidelines resembles the description in Finland, where the central means of identifying and intervening in maltreatment include knowing and evaluating signs of child maltreatment, discussing the family situation and relationships with parents, discussing child-rearing practices, conducting home visits, and employing multiprofessional practices [27].

**Table 1:** Communication tools employed by PHNs in CFHCs regarding child maltreatment

***In Safe Hands*** a film designed and used at the CFHC to prevent child maltreatment.(English version at: https://www.youtube.com/watch?v=HyEVfoGWC2U)
***Parent Toolkit*** is an information tool designed to support parents. (English version at: https://www.10smartetips.no/en/)
***Understanding of our body and sexuality – Empowering Toddlers*** a brochure about 2-year-old child's understanding of their body and sexuality
***Language4*** a language assessment tool originally designed to evaluate language development in children aged 4–6 years. The tool consists of picture naming, sentence formation, story retelling, and sound discrimination. Språk 4 is widely used in educational and clinical settings in CFHC

The duty of confidentiality for PHNs is stipulated in the Health Personnel Act, which allows for exceptions in cases where factors could harm a child is present. Corporal punishment is illegal in Norway [30], encompassing hitting (e.g. slapping or spanking, with a hand or an object) and other forms of physical harm (e.g. pinching, biting, pulling hair). PHNs are obligated reporting to Child Protective Service (CPS) if there is a *'reason to believe that the child has been or is at risk of experiencing*' maltreatment and/or witnessing parental intimate partner violence (IPV) [28]. PHNs must also assess whether there are grounds for reporting to other emergency services, including the police. The responsibility of CPS is to further investigate the case and implement necessary measures [31].

Even though attendance at CFHC check-ups are voluntary, children have the legal right to health assessments [32], and the service has high coverage. Nearly all children and families (99%) adhere the recommended program and visits at their CFHC [33]. Thus, PHNs, who interact frequently with families during early childhood, are uniquely positioned to early prevention, and detection of child maltreatment. However, a white paper (NOU, 2017, p. 12) that examined cases of child maltreatment in Norway stated that children were overlooked in many cases where the maltreatment should have been stopped earlier [34]. In 2019, the National Criminal Investigation Service in Norway (KRIPOS) released a report on severe cases of maltreatment of children aged 0–4 years. The investigation found that more than 70% of cases involved infants less than one year old, with 50% occurring in children younger than five months old [35]. These children are not yet enrolled in daycare. Consequently, PHNs may be their primary non-family contacts, highlighting the significant role and responsibility of PHNs during this period of infancy.

Previous research has indicated that nurses working in primary care acknowledge the significance of detecting child maltreatment, but also recognize the challenges associated with the task [36, 37]. Difficulties in communication and identifying less obvious forms of maltreatment contribute to these challenges [38–41]. According to a systematic review conducted by Wilson and Lee (2021), there are multiple factors that can hinder the reporting of incidents. These barriers can stem from structural limitations, resource limitations, insufficient support systems, sociocultural influences, and individual characteristics [39]. There is limited research on how healthcare professionals in primary care comprehend and implement guidelines for preventing and detecting child maltreatment, and quantitative studies are lacking. A recent systematic review on the public health approach on child maltreatment, and adverse childhood experiences, underscores the urgent need for more research using a public health perspective [41].

## Aim

The objective of this study is to describe how PHNs at CFHCs follow the National Clinical Guidelines to prevent and detect child maltreatment, with a particular focus on clinical procedures and interdisciplinary collaboration. Furthermore, we aim to determine factors that are associated with identification of child maltreatment.

## Methods

### Design and sample

A cross-sectional survey was employed to collect data among PHNs working at a CFHC between October 24th and December 31st, 2022. Norwegian PHNs are registered nurses with additional education in health promotion and preventive care, specialized in children's psychological, physical, and emotional development [42]. PHNs who worked with children aged 0–5 years at the CFHC and had consultations with families were eligible for participation. The PHNs were recruited from the largest and only professional association for PHNs in Norway, the register of the Norwegian Union of Public Health Nurses. An electronic questionnaire was distributed to all members who had a valid email address, resulting in a total of 3798 out of 3896 members who received the email (97.5%). It should be noted that there are 98 PHNs (2.5%) missing due to unavailable email addresses. All members were encouraged to respond, as we did not know who worked in CFHC or other parts of the service. Reminder emails were sent to enhance response rates after 9 days, 3 weeks, and 7 weeks. To gain an understanding of the population and sample included in the study, demographic inquiries such as age, geographic location, population size, education, and experience were incorporated into the survey. Question seven specifically inquired whether the PHNs worked at a CFHC, with exclusions applied to those who worked in other areas of the service, such as PHNs at schools, infection control, migration and so on. Initially, 1873 PHNs responded to the survey. Among them, 1238 were excluded because they did not work in a CFHC, and an additional 45 were excluded because they did not participate in family consultations. The final analysis included responses from 554 PHNs who worked in CFHC and participated in family consultations.

### Ethical considerations

Approval was obtained from the Norwegian Service for Shared Service in Education and Research (SIKT), registration number 303782. The study information sheet was distributed along with a link to the survey. Informed consent was indicated by questionnaire completion; no identifiable information was collected.

Excluded participants were accordingly notified of their ineligibility and presented the opportunity to withdraw. In recognition of the delicate nature surrounding the topic of child maltreatment, no questions regarding this topic were posed to the PHNs who were not included in the study.

### Questionnaire

**Fig. 1:**
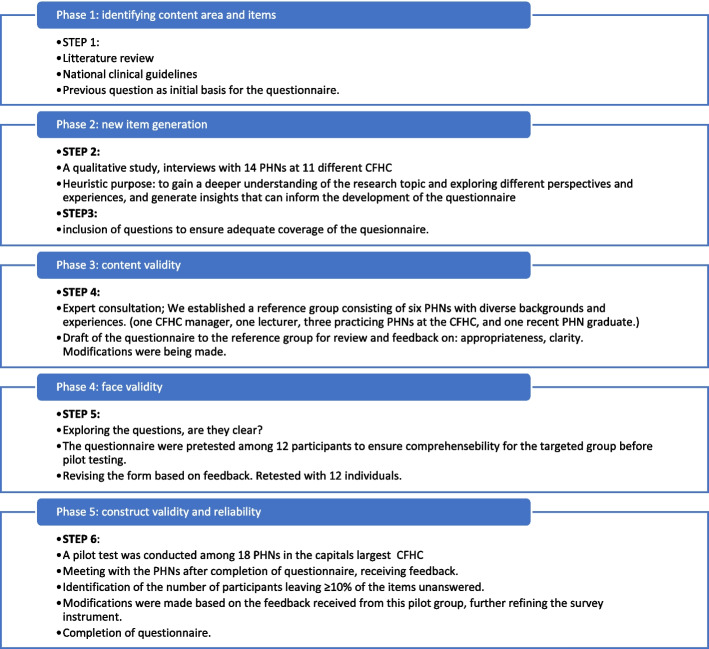
Development process of the questionnaire

Due to the absence of an existing, suitable, and validated questionnaire, a survey instrument was designed for this study to address the specific research objectives as part of the project: ‘Public Health Nurses in Child and Family Health Clinics role in preventing and detecting child maltreatment’. The development process of the questionnaire followed a modified five-phase approach (Fig. 1) adapted from Streiner, Norman, and Carineys [43]. The questionnaire construction drew upon existing studies [44–47] and the National Clinical guidelines for PHNs within CFHC settings [28]. To ensure questionnaire validity, a qualitative study gathered insights from PHNs in CFHC on detecting and preventing child maltreatment during the development process of the questionnaire [48]. The completed survey comprised a total of 128 questions and 46 sub-questions, organized into five sections including demographics, guidelines and experience of child maltreatment, vignettes, knowledge and attitude (Supplementary file 1). It included both closed-ended and open-ended response options. This article will address questions related to PHNs' procedures and interdisciplinary collaboration in their clinical efforts to prevent and detect child maltreatment. Subsequently, we will address questions about PHNs' experiences of suspecting child maltreatment at the CFHC and the frequency of these suspicions have occurred.

Demographic variables, including age, geographic location, population size, education, employment status and years of experience were collected. Additionally, we investigated the organization of CFHCs including staff and size. We used categorical response options to protect anonymity and excluded gender due to low male representation. We assessed years of experience working in CFHCs and the number of PHNs at each CFHC. Furthermore, we explored the presence of other professions at CFHCs, such as doctors, midwives, and therapists.

Questions about the procedures in detecting and preventing child maltreatment, we followed the description outlined in CFHC guidelines. The response options regarding procedures were based on the recommendations and indicated whether a procedure should be universally implemented at each consultation, carried out during specific/targeted consultations (for example, parental education about Shaken Baby Syndrome at the first postnatal visit), or based on indications when presence of signs, symptoms, or information indicating risk. Due to findings from the qualitative study conducted prior to the survey [48], which revealed that PHNs had more experience in identifying emotional maltreatment and neglect compared to physical violence, we opted for specific yes/no questions for each type of child maltreatment to measure PHNs' experience in detecting child maltreatment at the CFHC. "Have you ever encountered any children in your work at the CFHC whom you suspected being a victim of physical violence? Yes/No." Those responding affirmatively were further asked about the frequency of such instances a year.

Interdisciplinary collaboration questions were based on the content of the guidelines and PHNs statutory obligation to report to CPS. Given the predominantly mandatory nature of most survey questions, we included 'don't know' or 'prefer not to answer,' options for many of them.

Finally, we included six statements regarding collaboration. For response options to these assertions, we chose a 7-point Likert scale, ranging from "strongly agree" to "strongly disagree". The Likert scale is recognized for assessing levels of agreement/disagreement, and a 7-point scale is considered to be more sensitive than fewer alternatives [49].

### Data analysis

The analysis was performed using IBM SPSS Statistics software, Version 28.01.01. The summary of the data is presented as frequency, percentage and mean. We consolidated certain response options into larger groups if there were few responses within a category to address sparse data.

Relationship Analysis: To investigate potential associations between demographic variables (age, additional education beyond the PHN degree, years of experience in the CFHC, the size of the CFHC) associated with identification of child maltreatment, we performed a univariate logistic regression analysis. Due to a limited number of respondents in age category below 30 years, < 30 and 31–40 were combined. Responses 'Prefer not to answer' (0.2%) for age were recoded as missing before logistic regression analysis for data integrity and analytical rigor. In this analysis, the dependent variable was affirmative responses regarding experience in suspicion of child maltreatment. Significant variables from the univariate analysis were included in a multivariate regression analysis to control for potential mediator factors.

## Results

### Demographics

Table 2 presents demographic characteristics of the 554 respondents who completed the questionnaire. Almost all PHNs (97.5%) were registered nurses with specialization as PHNs. Most respondents (57.2%) worked over 80% of full-time hours. The levels of experience working in CFHCs varied, with the largest group (43.3%) having more than 10 years of experience, followed by 24.7% with 6–10 years of experience. The majority of PHNs worked in larger cities, 31.8% in areas with populations of 15,000 to 30,000, and 15.5% in rural areas with populations of fewer than 5,000 inhabitants. CFHCs typically had 2–5 PHNs (42.2%) and included other professions such as doctors (96.9%), midwives (88.8%), and secretarial staff (81.2%). Most of the PHNs (76.9%) reported carrying out the recommended 14 consultations, 15.9% most of the times, while 7.0% did not provide them.

**Table 2:** Demographic characteristics of the public health nurse respondents

**PHNs (N 554)**	n (%)
Age (years)	
< 30–40	143 (25.8)
41–50	208 (37.5)
51–60	139 (25.1)
> 60	63 (11.4)
Don’t wish to answer	1 (.2)
What region in Norway do you work?	
Northern region	64 (11.6)
Central region	40 (7.2)
Western region	138 (24.9)
Eastern region	240 (43.3)
Southern region	72 (13.0)
Number of inhabitants where the CFHC^a^ is located	
< 5000	86 (15.5)
5000–10.000	61 (11.0)
10.000–15.000	57 (10.3)
15.000–30.000	119 (21.5)
> 30.000	214 (38.6)
Don’t know	17 (3.1)
Are you educated as an RN^b^ with PHN^c^ degree?	
Yes	539 (97,3)
No	9 (1,6)
Currently undergoing education for PHN	6 (1,1)
Other/additional education/qualifications than a PHN	
Yes	217 (39.2)
% of full-time post	
< 20	34 (6.1)
20–50	67 (12.1)
50–80	136 (24.5)
> 80	317 (57.2)
Experience working in CFHC (in years)	
< 2	57 (10.3)
2–5	120 (21.7)
6–10	137 (24.7)
> 10	240 (43.3)
How many PHNs work at the CFHC	
1 (only me)	24 (4.3)
2–5 PHNs (small CFHC)	234 (42.2)
6–10 PHNs (Medium CFHC)	171 (30.9)
> 10 PHNs (Large CFHC)	125 (22.6)
Other professions at the CFHC	
CFHC-doctor	537 (96.9)
Midwife	492 (88.8)
CFHC receptionist	450 (81.2)
Auxiliary nurses/staff	64 (11.6)
Physiotherapist	419 (75.6)
Occupational therapist	115 (20.8)
Other^d^	133 (24.0)
Does the CFHC provide and carry out the 14 consultations recommended in the guidelines?	
Yes	426 (76.9)
No	39 (7.0)
Most of the times	88 (15.9)
Don’t know	1 (.2)

^a^Child and Family Health Clinic

^b^Registered Nurse

^c^public health nurse

^d^The answer lead to a follow-up open-ended question asking ‘which other professions?’ (not included in current study)

### Procedures to detect and prevent child maltreatment

When assessing for risk factors/stressors in the family, 43.0% of PHNs assessed at every CFHC visit, 42.2% assessed during specific/targeted consultations (Table 3). Additionally, 14.6% assessed based on indications. Nearly all PHN (93.7%) provided information about child maltreatment during specific/targeted consultations, with some doing so in every consultation (2.7%) or based on indications (3.4%). Less than one percent never engaged in such discussions. Discussions about setting boundaries, discipline, and corporal punishment mainly occurred during specific/targeted consultations (61.7%), based on indications (29.8%), with a few having these conversations at every consultation (4.9%).

**Table 3:** PHNs^a^ procedures in preventing and detecting child maltreatment (*N* 554)

In my effort to prevent or detect child maltreatment	n (%)
I assess for risk factors/stressors in the family at (*n* = 554)	
Every consultation	238 (43.0)
Specific/targeted consultations	234 (42.2)
Based on indications	81 (14.6)
Don’t know	1 (.2)
I ask the parents about their own childhood at (*n* = 554)	
Every consultation	4 (.7)
Specific/targeted consultations	429 (77.4)
Based on indications	110 (19.9)
Never	10 (1.8)
Don’t know	1 (.2)
I teach/guide the parents about child maltreatment (eg. Sbs^b^, persistent crying and risk factors) at (*n* = 554)	
Every consultation	15 (2.7)
Specific/targeted consultations	519 (93.7)
Based on indications	19 (3.4)
Never	1(.2)
I talk about genital mutilation at (*n* = 554)	
Every consultation	1 (.2)
Specific/targeted consultations	191 (34.5)
Based on indications	309 (55.8)
Never	47 (8.5)
Don’t know	6 (1.1)
I have conversations about setting boundaries, discipline and corporal punishment (*n* = 554)	
Every consultation	27 (4.9)
Specific/targeted consultations	342 (61.7)
Based on indications	165 (29.8)
Never	16 (2.9)
Don’t know	4 (.7)
I assess the child’s well-being and weight at (*n* = 554)	
Every consultation	542 (97.8)
Specific/targeted consultations	5 (.9)
Based on indications	7 (1.3)
I assess the child's physical development at (*n* = 554)	
Every consultation	513 (92.6)
Specific/targeted consultations	22 (4.0)
Based on indications	10 (1.8)
Never	6 (1.1)
Don’t know	3 (.5)
I assess the child's social development at (*n* = 554)	
Every consultation	540 (97.5)
Specific/targeted consultations	7 (1.3)
Based on indications	6 (1.1)
Don’t know	1 (.2)
Do you provide extra consultations? (*n* = 554)	
Yes^c^	553 (99.8)
^c^Approximately, how many extra consultations do you have a week? (*n* = 553)	
< 2	118 (21.3)
2–5	374 (67.6)
> 6	41 (7.4)
Don’t know	20 (3.6)
Do you ever get contacted by parents outside of working hours as a PHN? (*n* = 554)	
Yes^d^	351 (64.4)
Do you use instruments, such as brochures, film etc. in conversation about child maltreatment with parents/child?^e^ (*n* = 554)	
Yes	553 (99.8)

^a^Public health nurse

^b^Shaken Baby Syndrome

^c^Triggered follow-up question/the follow up question

^d^Triggered the follow-up question In what way? (not in table)

^e^The question has been revised for international comparability. The Likert scale responses were consolidated into 'yes,' and 'do not use' was categorized as 'no.' Details on the tools is presented in Box1

Furthermore, almost all PHNs offered additional consultations (99.8). All PHNs except one utilized tools, such as brochures and films during discussions on child maltreatment, with the film ‘In Safe Hands’ being the most used and considered the most effective tool by 91.7% of PHNs (Supplementary Table 1). Additionally, 36.6% of PHNs reported being contacted by parents outside of their regular working hours. Parents reached out through various means, including work phones (46.9%), private phones (22.8%), social media (13.2%), or email (6.2%) (not in tables).

### Interdisciplinary collaboration

A total of 31.8% contacted general practitioners (GPs), mostly infrequently (< 1/year) when suspecting child maltreatment (Table 4). Similarly, referral to specialist healthcare services was reported by 35.4%, with only 4.6% doing so frequently. A total of 17.0% had contacted the police, primarily about physical violence (73.4%). A significant number of PHNs (71.1%) in our study had prior experience of filing reports to the CPS. The distribution of these reports was even across various types of maltreatment, with neglect being identified as the most prevalent cause (75.5%). More than 80% of respondents reported rarely or never being invited for collaboration meetings with CPS following the submission of their concerns. The PHNs reported infrequent invitations for collaboration initiated from others with concerns about a child. In the statements about collaboration with the 7-point Likert scale (Supplementary Table 2), it was evident that collaboration was rated high between midwives (6.1, 1.18) and PHNs, as well as other professions (6.0, 1.03) within the CFHC. However, these ratings exhibited a decline in the case of collaborative interactions statements involving CPS (4.2, 1.65).

**Table 4:** Interdisciplinary collaboration when suspecting child maltreatment

	n (%)
**Have you ever contacted a GP**^**a**^** with concerns of child maltreatment? (*n* = 554)**	
Yes	176 (31.8)
**How often? (*n* = 176)**	
< 1 a year	124 (70.5)
1–2 times a year	44 (25.0)
3 times or more	6 (3.4)
Missing	2 (1.1)
**Have you ever contacted a specialist healthcare service (e.g.. pediatric department) with concerns of child maltreatment? (*n* = 554)**	
Yes	196 (35.4)
**How often? (*n* = 196)**	
< 1 a year	116 (59.2)
1–2 times a year	68 (34.7)
3 times or more	9 (4.6)
Missing	1 (.5)
**Have you ever reported concerns of child maltreatment to the police?**	
Yes	94 (17.0%)
**With what type of concerns? (*n* = 94)**	
Physical Violence	69 (73.4)
Psychological/emotional violence	33 (35.1)
Neglect	13 (13.8)
Witnessing parental IPV^c^	43 (45.7)
Sexual abuse	44 (46.8)
**Have you ever reported concerns of child maltreatment to the CPS**^**b**^**? (*n* = 554)**	
Yes	397 (71.7)
**With what type of concerns? (*n* = 397)**	
Physical Violence	286 (72.0)
Psychological/emotional violence	275 (69.3)
Neglect	300 (75.5)
IPV	265 (66.8)
Sexual violence	119 (30.0)
**In cases where you have filed a report to CPS. How often are you called in for a collaborative meeting about the family by CPS? (*n* = 397)**	
Never	143 (36.0)
Rarely	198 (49.9)
Nearly every time	52 (13.1)
Allways	3 (.8)
Missing	1 (.3)
**How often are you invited for collaboration by the following entities regarding concerns for a child? (*n* = 554)**	
**GP**	
**< **1 a year or never	480 (86.6)
1–2 times a year	53 (9.6)
> 3 times a year	21 (3.8)
**CPS**	
**< **1 time a year or never	287 (51.8)
1–2 times a year	58 (10.5)
> 3 times a year	8 (1.4)
**Police**	
**< **1 time a year or never	539 (97.3)
1–2 times a year	14 (2.5)
> 3 times a year	1 (.2)
**Hospital/children ward**	
**< **1 time a year or never	488 (88.1)
1–2 times a year	58 (14.1)
> 3 times a year	8 (1.4)

^a^general practitioner

^b^Childrens Protective Service

^c^Intimate Partner Violence

### Experience and factors influencing PHNs' suspicions of child maltreatment

Table 5 presents the findings regarding PHNs experiences in suspecting different forms of child maltreatment at CFHCs. The majority of PHNs reported encountering cases involving suspicions of all various types of maltreatment.

**Table 5:** PHNs^a^ experience of suspecting child maltreatment. (*N* = 544)

**Have you encountered any children in your work at the CFHC**^**b**^** whom you suspected of being victims of**	n (%)
**Physical violence (*n* = 554)**	
Yes	391 (70.6)
**How often do you encounter children at CFHC where you suspect physical violence? (*n* = 391)**	
< 1 a year	159 (40.7)
1–2 a year	165 (42.5)
> 3 a year	55 (14.1)
Don’t know/remember	12 (13.1)
**Psychological/emotional violence (*n* = 554)**	
Yes	465 (83.9)
**How often do you encounter children at CFHC where you suspect emotional/psychological violence? (*n* = 465)**	
< 1 a year	84 (18.1)
1–2 a year	217 (46.7
> 3 a year	149 (32.0)
Don’t know/remember	15 (3.2)
**neglect (*n* = 554)**	
Yes	452 (81.6)
**How often do you encounter children at CFHC where you suspect neglect? (*n* = 452)**	
< 1 a year	172 (38.1)
1–2 a year	193 (42.7)
> 3 a year	74 (16.4)
Don’t know/remember	13 (2.9)
**witnessing parental IPV**^**c**^** (*n* = 554)**	
Yes	428 (77.3)
**How often do you encounter children at CFHC where you suspect witnessing parental IPV? (*n* = 428)**	
< 1 a year	149 (34.8)
1–2 a year	202 (47.2)
> 3 a year	60 (14.0)
Don’t know/remember	15 (3.5)
Missing	2 (.4)
**Sexual violence (*n* = 554)**	
Yes	190 (34.3)
**How often do you encounter children at CFHC where you suspect sexual violence? (*n* = 190)**	
< 1 a year	143 (75.3)
1–2 a year	40 (21.1)
> 3 a year	2 (1.1)
Don’t know/remember	5 (2.6)

^a^Public Health Nurse

^b^Child and Family Health Clinic

^c^Intimate Partner Violence

Among the PHNs who reported having suspicions, the majority expressed experiencing infrequent encounters with all the different types of maltreatment, with such incidents typically occurring less than once a year. There were variations in the reported frequency, with some PHNs reporting more frequent encounters. Psychological/emotional violence was the most frequent suspected form of maltreatment, with 149 PHNs (32.2%) reporting encounters with more than three children per year where they suspected this form of maltreatment.

In the logistic regression analysis presented in Table 6, the association between age and suspecting physical violence remained significant in the multivariate logistic regression for PHNs over 61 having higher odds (AOR: 2.59, CI: 1.07–6.28). PHNs under the age of 40 demonstrated higher odds of suspecting witnessing parental IPV compared to their older counterparts (AOR: 1.86 95% CI: 1.05–3.31). Years of experience in CFHC also exhibited significant associations, with PHNs having less than 2 years of experience showing lowered odds in all the different types of child maltreatment. Additionally, having additional education was associated with increased odds of suspecting sexual violence (AOR: 1.71, CI: 1.16–2.52). Employment status and the number of PHN colleagues in CFHC did not show significant associations with any type of child maltreatment in the multivariate analysis, except from suspicions of neglect and sexual violence where PHNs in medium CFHC had reduced odds.

**Table 6:** Logistic regression of public health nurses in child and family clinics suspecting child maltreatment

Multivariate logistic regression	Physical violence			Psychological violence			Neglect			Witnessing parental IPV			Sexual Violence		
	OR	95% CI	***p***-value	OR	95% CI	***p***-value	OR	95% CI	***p***-value	OR	95% CI	***p***-value	OR	95% CI	***p***-value
**Age (in years)**															
< 40	1.22	.74–2.03	.438	1.15	.61–2.17	.673	.808	.45–1.44	.468	1.86	1.05–3.31	.035	1.33	.74–2.39	.345
41–50 (ref)															
51–60	1.02	.59–1.74	.954	.49	.24–1.02	.057	.870	.42–1.79	.705	1.16	.61–2.21	.649	2.12	1.30–3.46	.003
> 61	2.59	1.07–6.28	.035	.75	.26–2.14	.594	.840	.30–2.35	.739	.92	.40–2.14	.846	2.55	1.35–4.82	.004
**Additional education/qualifications**															
Yes													1.71	1.16–2.52	.007
No (ref)															
**Employment status (%)**															
< 20				.39	.16–98	.046									
20–50				.69	.31–1.53	.365									
50–80				.52	.29-.91	.023									
> 80 (ref)															
**Years of experience in CFHC**															
< 2	.16	.08-.34	< .001	.06	.02-.14	< .001	.036	.03-.15	< .001	.05	.02-.11	< .001	.25	.09-.61	< .001
2–5	.38	.21-.69	.001	.20	.09-.45	< .001	.242	.11-.53	< .001	.23	.12-.46	< .001	.26	.15-.51	.932
6–10	.73	.42–1.26	.254	.53	.24–1.17	.117	.321	.15-.67	.003	.48	.25-.92	.026	.98	.59-.1.62	.932
> 10 (ref)															
**How many PHN colleagues in CFHC**															
Small CFHC (ref)^a^															
Medium CFHC^b^							.39	.23-.67	< .001				.65	.42–1.02	.061
Large CFHC^c^							.93	.50–1.75	.829				.60	.36-.99	.047

^a^< 5PHNs in the CFHC

^b^6-10 PHNs in the CFHC

^c^> 10 PHNs in the CFHC

## Discussion

This study aimed to investigate how PHNs at CFHCs implement the National Clinical Guidelines in regards to prevention and identification of child maltreatment, focusing on clinical procedures and interdisciplinary collaboration. Additionally, we explored variables correlated with an elevated rate of child maltreatment identification.

The PHNs in this study largely adhere to the recommended procedures described in the guidelines. They routinely screen for child maltreatment, assess familial risk factors, provide preventive measures such as anticipatory guidance and educate parents about child maltreatment, and offer additional follow-up when needed. Only a few PHNs reported to never talk about discipline and corporal punishment.

These findings are consistent with the qualitative study conducted among PHNs in CFHC prior the survey, where the PHNs at CFHC systematically and deliberately integrated efforts to prevent and detect prevent child maltreatment as part of their regular practice [48]. Activities such as ongoing mapping and assessment of risk factors, educating parents about child maltreatment (eg. shaken baby syndrome) and tailoring follow-up interventions based on individual needs were part of this endeavor. Moreover, other studies support PHNs adherence to the guidelines, although some disparities in engagement with preventive procedures (27, 01). In Eija et al., 61% of the PHNs reported assessing family risk factors, while 67% reported discussing discipline with parents [27]. In Suzuki et al., the study compared practices between Finland and Japan found that 72% of respondents in Finland engaged in discussions about risk factors, whereas only 54% of health nurses in Japan did so [50]. In that study, 93% of respondents from Finland agreed that the guidelines prompted changes in their work routines to align with recommendations, indicating a strong commitment to adherence. Conversely, in Japan, only 13% agreed, suggesting a significant challenge in aligning practices with the guidelines. It's worth noting that these studies were conducted prior to or shortly after the WHO European report focusing on child maltreatment prevention, which emphasizes the assessment of risk factors and parental support, as well as a low acceptance of corporal punishment [14]. The Norwegian guidelines were revised and updated in 2017, with the chapter on child maltreatment receiving enhanced focus on both prevention and detection [28], implying that Norwegian PHNs have had a longer period to integrate this change into their practice.

Nearly all of the PHNs in our study reported to provide additional consultations for children and more than half of PHNs reported being in touch with families outside of working hours. This contrasts with the findings from a study among healthcare professionals in pediatric departments in Norwegian hospitals, where there was little inclination to further follow up on children, despite concerns about child maltreatment [44]. Although our findings indicate a high level of availability and a strong commitment from PHNs, the Norwegian official reports contends that there are too few reports of child maltreatment and reported cases could have been detected earlier [34, 35]. The thorough follow-up described by the PHNs may reflect concerns and uncertainty, which is considered a barrier in reporting child maltreatment [27, 39, 48, 51]. A Danish study about management of child maltreatment suspicions in general practice found that GPs and practice nurses often felt left to themselves managing their suspicions, especially in cases without clear signs and emphasized the importance of scheduling new appointments as a strategy to track the child's progress and maintaining a relation to the child’s family [51]. The extensive follow-up may also indicate that PHNs are trying to address the child’s needs in other ways than reporting. Findings from a quantitative study indicated that primary care professionals did not fulfill their mandatory obligation diligently; instead, they suggested addressing it through alternative means to provide assistance [52]. In Midtsund et al.'s qualitative study, several PHNs reported negative experiences when reporting to CPS, such as CPS dismissing cases where the PHN had serious concerns and felt a strong commitment to follow up on the children [48]. The statistics from Statistics Norway (SSB) show a significant increase in the concern reports reviewed by CPS when children are older and done with the follow-up from the CFHC. In 2022, there was a 66.5% rise in the concern reports within the age group 6–12 years compared to children in the age group 0–5 years [53]. This shift raises important questions about the extent of support provided, whether PHNs are taking on excessive responsibility and prolonging their involvement or might illustrate the need for more knowledge about actions and decisions from CPS.

Many PHNs in our study had experience in suspecting child maltreatment and submitted reports of concern to CPS, but they were rarely invited for collaboration meetings. Several studies conducted in primary care settings describe the frustration experienced by healthcare professionals regarding children referred to CPS and the lack of information sharing about what happens [38, 48, 54, 55]. Confidentiality is frequently cited as a barrier [39]. However, two recent qualitative studies conducted within primary care among GPs and PHNs did not perceive confidentiality obligations as an obstacle to potential collaboration [48, 55]. Instead, they explained that a one-way flow of information and a lack of open dialogue perpetuated lack of knowledge about CPS and uncertainty about their family’s situations (ibid).

In addition to the lack of collaboration with CPS, our study revealed limited collaboration among various stakeholders within the health sector, including GPs and hospitals. These findings are consistent with prior research and lack of collaboration across disciplines [24–26]. Moreover, previous research suggests that detecting child maltreatment in primary healthcare settings, such as in CFHCs, can be challenging primarily because healthcare professionals often encounter less severe and acute clinical conditions, making it difficult to identify child maltreatment [40]. The majority of PHNs in our study have encountered situations where they suspected different forms of child maltreatment, with psychological/emotional violence and neglect being the most frequently reported. It became apparent in the follow-up question that suspicion of all forms of maltreatment was an uncommon occurrence. Physical violence was the second least suspected, and sexual violence even less. In Midtsund et al. study (2023) PHNs explained because CFHC check-ups are prescheduled, voluntary, and non-urgent, and therefore easier to cancel if there are bruises or marks needed to hide [48].

Concurrently, other research argues that diagnosing child maltreatment poses specific challenges for hospital healthcare professionals due to uncertainty [56]. Decisions must be made swiftly due to the potential harm to the child and the inherent uncertainties and ambiguities that cannot be entirely eliminated. Considering these findings, collaboration between primary healthcare and specialized healthcare settings is essential. PHNs, who have been involved in monitoring the child's development over time, assessing risk factors, and developing a deep understanding of the family dynamics, can contribute significantly to a more comprehensive assessment for hospital healthcare professionals who deal with acute injuries. Additionally, information from hospital admissions could provide a more holistic picture and potentially reinforce or alleviate existing concerns of PHNs regarding a child's well-being. Findings in a meta-synthesis of qualitative studies indicate that mandate reporters, as health care providers, struggle to identify and respond to less obvious forms of child maltreatment and more effective communication and information sharing would be of interest of all services for better assessments and support in child maltreatment [40].

In our study there was a notable difference in the incidence of suspicions of child maltreatment based on age and years of experience among PHNs in CFHCs. Our findings suggest that experience and age may influence the ability to recognize signs of physical violence. Older PHNs had higher odds of suspecting physical violence than younger PHNs, and PHNs with less than two years of experience reported less experience in suspecting maltreatment, in contrast to those with more experience. This is contrary to findings from a Finnish study among PHNs where neither age nor the number of years of experience as a nurse had a significant impact on the ability to identify or suspect child maltreatment [27]. Their study further showed that PHNs who had received training on child maltreatment demonstrated superior abilities in identifying maltreatment compared to their counterparts who lacked such experiences. In our study, however, additional education was found to be significant only in suspecting sexual violence. It is worth noting that the potential relationship between years of experience and suspicion of child maltreatment could be attributed to encountering a larger number of children in total. This exposure and accumulated experience may contribute to a sense of confidence in recognizing signs of maltreatment.

In a recent study conducted in Greece involving healthcare professionals from 60 hospitals, the findings revealed that older participants were more prone to suspect child maltreatment [57]. In contrast, we observed variations in the suspicion of child maltreatment across age groups and different forms of maltreatment. Specifically, we found an inverse relationship between age and suspicion of witnessing parental IPV, where PHNs under the age of 40 displayed a higher tendency to suspect witnessing parental IPV compared to the reference group. A possible explanation for this contrast could be that PHNs under the age of 40 received their PHN education more recently, after the 2017 update of guidelines that incorporated witnessing parental IPV as forms of child maltreatment.

### Strengths and limitations

This study demonstrates several strengths, including a large sample size with a wide geographic distribution. Although it is important to acknowledge that recruiting through the register of the Norwegian Union of Public Health Nurses may introduce concerns sampling bias, as membership may attract specific types of PHNs. Additionally, PHNs who participated in the study may have a particular interest in the subject matter, and those who do not adhere to guidelines may be less likely to participate. Not all PHNs in Norway are members of this Union, which may raise introduce concerns of generalizability.

The absence of non-union members in the study population challenges external validity, so caution should be exercised in generalizing the findings. Using internet questionnaires may introduce response bias, and the low number of participants in the oldest age group leads to wide odds ratios.

Another limitation is the absence of a suitable validated questionnaire, which raises concerns about data accuracy and validity. However, the careful step-by-step approach in questionnaire development, despite its lack of formal validation, can be considered a strength. Multiple tests and iterations were conducted to ensure validity, providing a strong foundation for data collection.

### Implications for practice

Research focusing on public health approaches to reduce child maltreatment is limited, and this study contributes to filling a knowledge gap in primary healthcare and offers valuable insights into the practices in of PHNs work regarding the detection and prevention of child maltreatment. This study provides a better understanding of Norwegian PHNs work regarding procedures and collaboration in primary care and CFHC setting. Additionally, the study confirms the lack of collaboration reported among various stakeholders, highlighting the need to address the issue and develop collaboration strategies to improve communication in the efforts to detection and prevention of child maltreatment.

The results can be utilized to develop and implement joint training for various professional groups. It is possible to foster practical collaboration through interdisciplinary training. The training should focus on familiarizing staff with the tasks of employees in other agencies/organizations, and on discussing what interdisciplinary collaboration means for each individual employee and for different professions. Training should also explore the prerequisites necessary for interdisciplinary collaboration and how it can be promoted.

## Conclusion

While PHNs in the study diligently adhere to the guidelines, suspicion of child maltreatment remains relatively uncommon. PHNs employ various clinical strategies in their efforts to detect and prevent child maltreatment. The study also outlines the lack of collaboration and highlights the need for improvement in this area. The findings further underscore the importance of fostering interprofessional collaborative skills, particularly among healthcare professionals such as hospital staff, GPs, and PHNs, as well as with CPS. This study provides a better understanding of the clinical procedures and assessments conducted by PHNs in the CFHC to prevent and detect child maltreatment. This insight fills a knowledge gap in the literature, providing increased understanding to other collaborators and thus facilitating better collaboration. The findings do not explain why there are too few cases of child maltreatment detected but provide insight into how PHNs implement the guidelines. Additionally, they suggest that age and experience may be influencing factors in the detection of child maltreatment. Although research on interventions aimed at preventing child maltreatment is challenging to demonstrate effectiveness, it is crucial to examine measures implemented for this purpose. This includes gaining knowledge about various tools described in the guidelines, such as the film ‘In Safe Hands’, from the perspectives of both parents and PHNs.

### Supplementary Information

Additional file 1: Supplementary Table 1: PHNs procedures preventing and detecting child maltreatment (N 554). Supplementary Table 2: Statements about collaboration (*N*=554)

Additional file 2: Supplementary file 1.

## Data Availability

The datasets generated and/or analyzed during the current study are not publicly available. This is to ensure participant confidentiality in accordance with current guidelines and laws. The datasets can be made available on reasonable request by contacting the corresponding author.

## Abbreviations

PHNs: Public Health Nurses
CFHC: Child and Family Health Clinic
GP: General practitioner
CPS: Child Protective Service

## References

[1] World Health Organization. Child maltreatment. Geneva: WHO; 2022. Available at: www.who.int/news-room/fact-sheets/detail/child-maltreatment.

[2] Graves K, Gay Y. Examining interpersonal violence from a trauma-informed and human rights perspective. In: Geffner R, White JW, Hamberger LK, Rosenbaum A, Vaughan-Eden V, Vieth VI. (eds) Handbook of interpersonal violence and abuse across the lifespan. Cham: Springer; 2022. 10.1007/978-3-319-89999-2_204.

[3] Segal L, Armfield JM, Gnanamanickam ES, Preen DB, Brown DS, dge J, et al. Child maltreatment and mortality in young adults. Pediatrics. 2021;147(1). 10.1542/peds.2020-023416. 10.1542/peds.2020-023416 33318224

[4] Stoltenborgh M, Bakermans-Kranenburg MJ, van Ijzendoorn MH, Alink LRA. Cultural-geographical differences in the occurrence of child physical abuse? A meta-analysis of global prevalence. Int J Psychol. 2013;48(2):81–94. 10.1080/00207594.2012.697165. 23597008 10.1080/00207594.2012.697165

[5] Irigaray TQ, Pacheco JB, Grassi-Oliveira R, Fonseca RP, Leite JC, Kristensen CH. Child maltreatment and later cognitive functioning: A systematic review. Psicologia Reflexão e Crítica. 2013;26:376–87. 10.1590/S0102-79722013000200018. 10.1590/S0102-79722013000200018

[6] Jaffee SR. Child maltreatment and risk for psychopathology in childhood and adulthood. Annu Rev Clin Psychol. 2017;13:525–51. 10.1146/annurev-clinpsy-032816-045005. 28375720 10.1146/annurev-clinpsy-032816-045005

[7] Strathearn L, Giannotti M, Mills R, Kisely S, Najman J, Abajobir A. Long-term cognitive, psychological, and health outcomes associated with child abuse and neglect. Pediatrics. 2020;146(4). 10.1542/peds.2020-0438. 10.1542/peds.2020-0438 PMC7786831 32943535

[8] Hughes K, Bellis MA, Hardcastle KA, Sethi D, Butchart A, Mikton C, et al. The effect of multiple adverse childhood experiences on health: a systematic review and meta-analysis. Lancet Public Health. 2017;2(8):e356–66. 10.1016/S2468-2667(17)30118-4. 29253477 10.1016/S2468-2667(17)30118-4

[9] Naughton AM, Maguire SA, Mann MK, Lumb RC, Tempest V, Gracias S, et al. Emotional, behavioral, and developmental features indicative of neglect or emotional abuse in preschool children a systematic review. JAMA Pediatr. 2013;167(8):769–75. 10.1001/jamapediatrics.2013.192. 23754198 10.1001/jamapediatrics.2013.192

[10] Harper NS. Child neglect: Evaluation and management. UpToDate 2023 https://www.uptodate.com/contents/child-neglect-evaluation-and-management.

[11] D’Arcy-Bewick S, Terracciano A, Turiano N, Sutin AR, Long R, O’Súilleabháin PS. Childhood abuse and neglect, and mortality risk in adulthood: A systematic review and meta-analysis. Child Abuse Negl. 2022;134:105922. 10.1016/j.chiabu.2022.105922. 36244209 10.1016/j.chiabu.2022.105922

[12] World Health Organization. Investing in children: The European child and adolescent health strategy 2015–2020. World Health Organization. 2014. Available from: https://apps.who.int/iris/handle/10665/337284WHO.

[13] Stoltenborgh M, Bakermans-Kranenburg MJ, Alink LR, van IJzendoorn MH. The prevalence of child maltreatment across the globe Review of a series of meta-analyses. Child Abuse Review. 2015;24(1):37–50. 10.1002/car.2353. 10.1002/car.2353

[14] World Health Organization. Regional Office for Europe. Investing in children: the European child maltreatment prevention action plan 2015–2020. World Health Organization. Regional Office for Europe; 2015. Available from: https://iris.who.int/handle/10665/350142.

[15] Hafstad GS, Sætren SS, Myhre MC, Bergerud-Wichstrøm M, Augusti E-M. Cohort profile: Norwegian youth study on child maltreatment (the UEVO study). BMJ Open. 2020;10(8):e038655-e. 10.1136/bmjopen-2020-038655. 32847921 10.1136/bmjopen-2020-038655 PMC7451483

[16] U.S. Department of Health and Human Services, Administration for Children and Families, Adminsitration on Children, Youth and Families, Children's Bureau. Child Maltreatment. 2020. Retrieved from: https://www.acf.hhs.gov/cb/data-research/child-maltreatment (2022).

[17] Bick J, Nelson CA. Early experience and brain development Wiley Interdisciplinary Reviews. Cogn Sci. 2017;8(1–2):e1387. 10.1002/wcs.1387. 10.1002/wcs.1387 PMC8103659 27906514

[18] Fox SE, Levitt P, Nelson CA III. How the timing and quality of early experiences influence the development of brain architecture. Child Dev. 2010;81(1):28–40. 10.1111/j.1467-8624.2009.01380.x. 20331653 10.1111/j.1467-8624.2009.01380.x PMC2846084

[19] World Health Organization. WHO guidelines on parenting interventions to prevent maltreatment and enhance parent–child relationships with children aged 0–17 years. Geneva; 2022. Available from: https://www.who.int/publications/i/item/9789240065505. 36810030

[20] Mikton C, Butchart A. Child maltreatment prevention: a systematic review of reviews. Bull World Health Organ. 2009;87(5):353–61. 10.2471/BLT.08.057075. 19551253 10.2471/BLT.08.057075 PMC2678770

[21] Harden BJ, Buhler A, Parra LJ. Maltreatment in infancy: A developmental perspective on prevention and intervention. Trauma Violence Abuse. 2016;17(4):366–86. 10.1177/1524838016658878. 27580663 10.1177/1524838016658878

[22] Child Welfare Information Gateway. Child maltreatment prevention: Past, present, and future. Issue Brief. 2017 1–17. https://www.childwelfare.gov/pubPDFs/cm_prevention.pdf.

[23] Inkilä J, Flinck A, Luukkaala T, Åstedt-Kurki P, Paavilainen E. Interprofessional collaboration in the detection of and early intervention in child maltreatment: employees' experiences. Nursing research and practice. 2013;2013. 10.1155/2013/186414. 10.1155/2013/186414 PMC3649739 23691298

[24] Cleek EA, Johnson NL, Sheets LK. Interdisciplinary collaboration needed in obtaining high-quality medical information in child abuse investigations. Child Abuse Negl. 2019;92:167–78. 10.1016/j.chiabu.2019.02.012. 30999166 10.1016/j.chiabu.2019.02.012 PMC6513678

[25] Herbert J, Ghan N, Salveron M, Walsh W. Possible factors supporting cross-agency collaboration in child abuse cases: a scoping review. J Child Sex Abus. 2021;30(2):167–91. 10.1080/10538712.2020.1856994. 33317439 10.1080/10538712.2020.1856994

[26] Backe-Hansen E, Smette I, Vislie C. Kunnskapsoppsummering. Vold mot barn og systemsvikt. Knowledge review. Violence against children and system failures. Oslo Metropolitan University - OsloMet: NOVA; 2017. Avaliable from: https://hdl.handle.net/20.500.12199/5110.

[27] Eija P, Mika H, Aune F, Leila L. How public health nurses identify and intervene in child maltreatment based on the national clinical guideline. Nurs Res Pract. 2014;2014. 10.1155/2014/425460. 10.1155/2014/425460 PMC4253703 25505986

[28] Norwegian Directorate of Health. Nasjonal faglig retningslinje for helsestasjons-og skolehelsetjenesten National guideline for health promotion and preventive work in the child and youth health clinics and school health service. 2019 Retrieved from: https://www.helsedirektoratet.no/retningslinjer/helsestasjons-og-skolehelsetjenesten/helsestasjon-05-ar.

[29] Norwegian Directorate of Health. Helsestasjon 0–5 år. Child family and health clinics 0–5 years. Available at: https://www.helsedirektoratet.no/retningslinjer/helsestasjons-og-skolehelsetjenesten.

[30] Barne- og familiedepartementet. Om lov om endringer i barnelova mv. (flytting, delt bosted, samvær, vold mv.) About the Act on Amendments to the Children's Act, etc. (relocation, shared residence, visitation, violence, etc.). Ot.prp.nr.104 2008–2009. Available from: https://www.regjeringen.no/no/dokumenter/otprp-nr-104-2008-2009-/id567744/?ch=6#fn1.

[31] Lov om barnevern (barnevernsloven) Child welfare act (LOV-1992–07–17–100), §2–2. Avaliable from: https://lovdata.no/lov/2021-06-18-97.

[32] Lov om pasient- og brukerrettigheter (LOV-1999–07–02–63), §6–1. Act on Patient and User Rights Avaliable from: https://lovdata.no/dokument/NL/lov/1999-07-02-63/.

[33] Statistics Norway, Activity in the Child Family Health Clinics and School Health Services, by healthcare consultations, statistical variable, year, and region. Table 11993. 2022. https://www.ssb.no/statbank/table/11993/tableViewLayout1/.

[34] NOU. Svikt og svik. Gjennomgang av saker hvor barn har vært utsatt for vold, seksuelle overgrep og omsorgssvikt Failure and betrayal. Review of cases where children have been exposed violence, sexual abuse and neglect. Barne- og Familiedepartementet. 2017 Available from: https://www.regjeringen.no/no/dokumenter/nou-2017-12/id2558211/.

[35] KRIPOS The National Criminal Investigation Service. (2019). Alvorlig vold mot små barn (1). Politiet Serious violence against young children (1). ThePolice. Available from: https://www.politiet.no/globalassets/04-aktuelt-tall-og-fakta/vold-mot-barn/alvorlig-vold-mot-barn.pdf.

[36] Dahlbo M, Jakobsson L, Lundqvist P. Keeping the child in focus while supporting the family: Swedish child healthcare nurses experiences of encountering families where child maltreatment is present or suspected. J Child Health Care. 2017;21(1):103–11. 10.1177/1367493516686200. 29156965 10.1177/1367493516686200

[37] Lines MLE, Grant JM, Hutton A. ‘How can we work together?’ Nurses using relational skills to address child maltreatment in Australia: A qualitative study. J Pediatr Nurs. 2020;54:1–9. 10.1016/j.pedn.2020.05.008. 32473486 10.1016/j.pedn.2020.05.008

[38] Engstrom M, Hiltunen J, Wallby T, Lucas S. Child Health Nurses’ experiences of addressing psychosocial risk factors with the families they meet. Acta Paediatr. 2021;110(2):574–83. 10.1111/apa.15492. 32716528 10.1111/apa.15492 PMC7891612

[39] Wilson IA, Lee J. Barriers and Facilitators Associated With Child Abuse and Neglect Reporting Among Child Care Professionals: A Systematic Review. J Psychosoc Nurs Ment Health Serv. 2021;59(6):14–22. 10.3928/02793695-20210324-01. 34060955 10.3928/02793695-20210324-01

[40] McTavish JR, Kimber M, Devries K, Colombini M, MacGregor JCD, Wathen CN, et al. Mandated reporters’ experiences with reporting child maltreatment: a meta-synthesis of qualitative studies. BMJ Open. 2017;7(10):e013942-e. 10.1136/bmjopen-2016-013942. 29042370 10.1136/bmjopen-2016-013942 PMC5652515

[41] Garrison ST, Gillen M, King LM, Cutshall K, Howitt A. Child maltreatment, adverse childhood experiences, and the public health approach: A systematic literature review. Int J Child Maltreat Res Policy Pract. 2022;5(4):541–71. 10.1007/s42448-022-00122-z. 10.1007/s42448-022-00122-z

[42] Utdanning.no. Public Health Nurse. Utdanning.no. Avaliable from: https://utdanning.no/yrker/beskrivelse/helsesykepleier.

[43] Streiner DL, Norman GR, Cairney J. Health measurement scales: a practical guide to their development and use. USA: Oxford University Press; 2015.

[44] Vollmer-Sandholm MJ, Myhre AK, Otterman G, Lydersen S, Stray-Pedersen A. Amongst Norwegian paediatricians there is little agreement in management of cases of suspected child maltreatment. Acta Paediatr. 2021. 10.1111/apa.16017. 34214215 10.1111/apa.16017

[45] Naughton A, Nurmatov U, Hoytema van Konijnenburg E, Watkins WJ, Otterman G, Nelson J, et al. Variability in Child Protection Medical Evaluations of Suspected Physical Abuse in Four European Countries: A Vignette Study. Child abuse review (Chichester, England : 1992). 2018;27(5):404–18. 10.1002/car.2515.

[46] Withiel TD, Gill H, Fisher CA. Responding to family violence: Variations in knowledge, confidence and skills across clinical professions in a large tertiary public hospital. SAGE open medicine. 2021;9. 10.1177/20503121211000923. 10.1177/20503121211000923 PMC7958155 33786184

[47] Fisher CA, Rudkin N, Withiel TD, et al. Assisting patients experiencing family violence: A survey of training levels, perceived knowledge, and confidence of clinical staff in a large metropolitan hospital. Women’s Health. 2020;16. 10.1177/1745506520926051. 10.1177/1745506520926051 PMC7385847 32716732

[48] Midtsund AD, Garnweidner-Holme L, Valla L, Lukasse M, Henriksen L. A qualitative study of public health nurses' experiences detecting and preventing child maltreatment in primary care settings. J Adv Nurs. 2023. 10.1111/jan.15761. 10.1111/jan.15761 37358075

[49] Taherdoost H. What Is the Best Response Scale for Survey and Questionnaire Design; Review of Different Lengths of Rating Scale / Attitude Scale / Likert Scale. Int J Acad Res Manag. 2022;8(1):1-. Available at: https://ssrn.com/abstract=4178693.

[50] Suzuki K, Paavilainen E, Helminen M, Flinck A, Hiroyama N, Hirose T, et al. Identifying and Intervening in Child Maltreatment and Implementing Related National Guidelines by Public Health Nurses in Finland and Japan. Nutr Res Pract. 2017;2017:5936781. 10.1155/2017/5936781. 10.1155/2017/5936781 PMC5317151 28265469

[51] Hoffmann Merrild C, Kjeldsen HC, Milidou I. Management of child maltreatment suspicions in general practice: a mixed methods study. Scand J Prim Health Care. 2023;41(1):81–90. 10.1080/02813432.2023.2178851. 37035862 10.1080/02813432.2023.2178851 PMC10088924

[52] Enosh G, Alfandari R, Nouman H, Dolev L, Dascal-Weichhendler H. Assessing, consulting, reporting heuristics in professional decision-making regarding suspected child maltreatment in community healthcare services. Child Maltreat. 2021;26(3):291–301. 32633613 10.1177/1077559520937351

[53] Statistisk sentralbyrå. Barn med bekymringsmelding etter alder Statistics Norway, Children with concern reports by age. Table 11292. 2022. Available from: https://www.bufdir.no/statistikk-og-analyse/barnevern/bekymringsmeldinger-til-barnevernet.

[54] Campbell KA, Wuthrich A, Norlin C. We Have All Been Working in Our Own Little Silos Forever: Exploring a Cross-Sector Response to Child Maltreatment. Acad Pediatr. 2020;20(1):46–54. 10.1016/j.acap.2019.06.004. 31185309 10.1016/j.acap.2019.06.004 PMC6899215

[55] Øverhaug OM, Laue J, Vis SA, Risør MB. ‘There’sa will, but not a way’: Norwegian GPs’ experiences of collaboration with child welfare services–a grounded theory study. BMC Primary Care. 2024;25(1):36. 38267870 10.1186/s12875-024-02269-9 PMC10807144

[56] Sigad LI, Beker G, Lev-Wiesel R, Eisikovits Z. “Alone with our interpretations”: Uncertainty in child abuse intervention among healthcare professionals. Child Youth Serv Rev. 2019;100:206–13. 10.1016/j.childyouth.2019.02.027. 10.1016/j.childyouth.2019.02.027

[57] Panagopoulou K, Gkentzi D, Fouzas S, Mentis M, Kostopoulou E, Plotas P, Papaevangelou V, Soldatou A. Child abuse experience, training, knowledge, and attitude of healthcare professionals in sixty hospitals in Greece. Eur Rev Med Pharmacol Sci. 2023;27(2). 10.26355/eurrev_202301_31068. 10.26355/eurrev_202301_31068 36734738

