# Supplementary material for: Detecting and preventing child maltreatment in primary care and PHNs’ role—a cross-sectional study
Source: BMC Prim Care. 2024 Jun 15;25:218. doi: 10.1186/s12875-024-02445-x (PMC11179210; doi:10.1186/s12875-024-02445-x)
Supplement: Supplementary file 1 — Additional file 1: Supplementary Table 1: PHNs procedures preventing and detecting child maltreatment (N 554). Supplementary Table 2: Statements about collaboration (N=554) [file 12875_2024_2445_MOESM1_ESM.docx]

| **Supplementary table 1**  **PHNs procedures preventing and detecting child maltreatment (N 554)** | Mean^1^ | SD |
| --- | --- | --- |
| **Question: How do you find the various tools to be effective as a starting point for discussing child maltreatment?** |  |  |
| 1. In Safe Hands [I trygge hender] (n=508) | 1.5 | .77 |
| 1. Parent Toolkit [Foreldrepakka] (n=411) | 1.6 | .68 |
| 1. Understanding of our body and sexuality – Empowering Toddlers   [Gode råd til deg som voksen når du snakker med barn om kropp og seksualitet] (n=287) | 2.2 | .86 |
| 1. Language4 [Språk4] (n=471) | 2.9 | 1.27 |

1: A 6-point Likert scale ranging, where 1 corresponds to 'excellent' and 6 corresponds to 'very poor’. (reponses: ‘doesn’t use’ was recoded into missing, in the analysis)

| **Supplementary table 2**  **Statements about collaboration (N=554)** | Mean^1^ | SD |
| --- | --- | --- |
| The collaboration between midwives and PHNs in cases of concern is good (n=554) | 6.1 | 1.18 |
| The collaboration between PHNs and other professions within the CFHC in cases of concern is good. (n=554) | 6.0 | 1.03 |
| The collaboration between PHNs and CPS is good (n=554) | 4.2 | 1.65 |
| I am invited to collaborative meetings with CPS when I have submitted a report of concern | 2.9 | 1.70 |
| I experience that the CPS dismiss cases of children who I am concerned. (n=554) | 4.6 | 1.45 |
| I receive discharge reports from hospitals when a child has been hospitalized. (n=554) | 5.0 | 1.70 |
| Confidentiality limits collaboration with others. (n=554) | 4.9 | 1.54 |

1: A 7-point Likert scale, where 1 corresponds to 'strongly disagree' and 7 corresponds to 'strongly agree.
