# Supplementary material for: Detecting and preventing child maltreatment in primary care and PHNs’ role—a cross-sectional study
Source: BMC Prim Care. 2024 Jun 15;25:218. doi: 10.1186/s12875-024-02445-x (PMC11179210; doi:10.1186/s12875-024-02445-x)
Supplement: Supplementary file 2 — Additional file 2: Supplementary file 1. [file 12875_2024_2445_MOESM2_ESM.pdf]

This is an English translation of the questions from the original Norwegian survey: *The Public Health Nurse's Work on Child Maltreatment 2022*. We would like to emphasize that the presentation will be different in a PDF format compared to the original digital format. The questions used in the present article are highlighted in blue.

The Public Health Nurse's Work on Child Maltreatment 2022

This is a survey about Public Health Nurse (PHN)s work with child maltreatment. We hope that you will take the time to respond. It takes approximately 35 minutes to complete the survey, but please use the time you prefer. (NOTE: Unfortunately, it is not possible to save your answers halfway through. If you close the survey before completing all the questions, you will need to start the survey again.) Responses or information collected will not be able to identify individuals in project reports, articles, or any potential master's theses. By answering the questions below, we consider that you consent to participate and to allow us to use your responses for research purposes.

Background information

What is your age?

- Under 30 years
- 31 - 40 years
- 41 - 50 years
- 51 - 60 years
- 61 years or older
- Prefer not to answer

Which County do you work in?

- Agder
- Innlandet
- Møre og Romsdal
- Nordland
- Oslo
- Rogaland
- Vestfold og Telemark
- Troms og Finnmark
- Trøndelag
- Vestland
- Viken
- Prefer not to answer

Number of inhabitants in the municipality/district where you work as a PHN?

- About 2000 or fewer inhabitants
- About 2000 - 5000 inhabitants
- About 5000 - 10000 inhabitants
- About 10000 - 15000 inhabitants
- About 15000 - 30000 inhabitants
- Over 30000 inhabitants
- Don't know

Are you educated as registered nurse (RN) with PHN degree?

- Yes
- No

Abbreviations: 1:PHN-public health nurse, 2:RN-registered nurse, 3:CFHC-child and family health clinic, 4:CPS-child protective service, 5:GP- general practitioner.

Currently pursuing education
Not applicable

How long ago did you graduate as a public health? (This question is only shown if "Yes" is selected in the question "Do you have a further education as a public health?")

Less than 1 year ago
1-2 years ago
3-5 years ago
6-10 years ago
11-15 years ago
More than 15 years ago

Do you have any other additional education/qualifications than PHN? (E.g. Pediatric nurse, midwife, family therapist, master's degree, etc.)

Yes
No

Which ones? (This question is only shown if "Yes" is selected in the question "Do you have any other additional qualifications?")

Are you working in Child and Family Health Clinic (CFHC) for ages 0-5?

Yes
No

What is your employment percentage? (This question is only shown if "Yes" is selected in the question "Are you working in CFHC for ages 0-5?")

10%
20%
30%
40%
50%
60%
70%
80%
90%
100%
Other

Comment: (This question is only shown if "Other" is selected in the question "What is your position at the CFHC?"

Are you in a position where you have consultations with children and families? (This question is only shown if "Yes" is selected in the question "Are you working in the CFHC for ages 0-5?")

Yes
No

This survey aims to investigate the work of PHNs in addressing child maltreatment among those who have consultations. If you are a professional supervisor or leader at the CFHC, we kindly ask you to encourage everyone at the CFHC to participate in this survey. Thank you very much for your help and participation! Please press send to complete the questionnaire so that we can include all respondents in the results.
Any comments: (This question is only shown if "No" is selected in the question " Are you in a position where you have consultations with children and families? ")

Abbreviations: 1:PHN-public health nurse, 2:RN-registered nurse, 3:CFHC-child and family health clinic, 4:CPS-child protective service, 5:GP- general practitioner.

If no, which part of the service do you work in? (This question is only shown if "No" is selected in the question "Are you working in the CFHC for ages 0-5?")

School health service
Secondary education
HFU (Youth and adolescent clinic)
Infection control (as a separate position)
Not working as a PHN
Not working/other/retired

(The following questions is only shown if "Yes" is selected in the question "Are you in a position where you have consultations with children and families?")

How many years have you worked at the CFHC for ages 0-5 in total?
Less than 2 years
Between 2 and 5 years
Between 6 and 10 years
More than 10 years

How many newborns (approximately) do you have in your position per year?

How many PHNs work at your CFHC? (This refers only to the number of employees in PHN positions. Not other personnel such as midwives, doctors, etc.)
1 (only me)
2-5
6-10
11-15
16-20
Over 20

What other professional groups work at/are employed at the CFHC? (Multiple selections possible)
CFHC doctor
Midwife
Secretary/administrative staff
Physiotherapist
Occupational therapist
Breastfeeding support counselor
Other

Please specify the other professional groups: (This question is only shown if "Other" is selected in the question "What other professional groups work at/are employed at the CFHC?")

Does the CFHC have a PHN responsible for professional development? (This refers to a PHN who has specific responsibility for updating and professional development for employees)
Yes
No
Don't know

CFHC Services for ages 0-5

Does the CFHC offer and conduct all 14 consultations in the CFHC program?
Yes

Abbreviations: 1:PHN-public health nurse, 2:RN-registered nurse, 3:CFHC-child and family health clinic, 4:CPS-child protective service, 5:GP- general practitioner.

No
Most of the time
Don't know

Check the consultation(s) that the CFHC potentially omits (Multiple answers possible) (This question is only shown if "No or Most of the time" is selected in the question "Does the CFHC offer and conduct all 14 consultations in the CFHC program?")

Home visit/first meeting
4 weeks
6 weeks with a doctor
6 weeks with a PHN
3 months
4 months
5 months
6 months with a doctor
6 months with a PHN
8 months
10 months
12 months with a doctor
12 months with a PHN
15 months
17-18 months
2 years with a PHN
2 years with a doctor
4 years with a PHN

Do you offer additional consultations?

Yes
No

How many (approximately) extra consultations do you have per week? (This question is only shown if "Yes" is selected in the question: Do you offer additional consultations?")

Less than 2 per week
Between 2-5 per week
Between 6-10 per week
Over 10 per week
Don't know

Do you ever get contacted by parents outside regular working hours as a PHN?

Yes
No

In what way? (Multiple answers possible) (This question is only shown if "Yes" is selected in the question "Are you sometimes contacted by parents outside of working hours?")

My private phone
My work phone
Visit my private home
Randomly meet after working hoursduring my free time
Messages on social media (Facebook, Instagram, etc.)
E-mail, which I respond to outside of working hours
Other ways

Abbreviations: 1:PHN-public health nurse, 2:RN-registered nurse, 3:CFHC-child and family health clinic, 4:CPS-child protective service, 5:GP- general practitioner.

Which other ways? (This question is only shown if "Other ways" is selected in the question "In what way?" Please specify the other ways.)

Do the children and families have a "designated PHN" at the CFHC? (A designated PHN means that the child has a person whom they (usually) meet every time they come to the CFHC)

- Yes
- No
- Don't know

To what extent is this realistic in practice? (This question is only shown if "Yes or Don't know" is selected in the question "Do you have a regular PHN at the CFHC?")

- To a very large extent
- To a large extent
- To some extent
- To a small extent
- To a very small extent
- Not at all
- Don't know

Do you have any comments on the question? (Please do not include personal information in the free-text area)

Vignettes – Management of difficult cases in CFHC

You will now be presented with three cases from a CFHC service. There are no right or wrong answers to these, but we want you to answer the questions based on what you believe would do if you were in these situation in practice.

Case 1:
A child comes for a four-year check-up with their mother. The PHN has been following the child since birth and has not had any previous concerns related to the child or the family. The child is shy at the beginning of the consultation, and the PHN must perform measurements of weight and height while the child holds the mother's hand. The child is wearing a T-shirt and underwear. The mother says that the child seems satisfied in daycare and has friends there. The child had steady growth up until the age of 2. The weight has increased significantly since the last check-up and crosses two percentiles for weight in relation to age. The height remains stable compared to previous measurements. The mother reveals that she and the child's father have separated since the last visit and that it has been a challenging period. The child becomes restless when the mother talks about this. During language assessment, the PHN asks what the child or the parents do when they get angry, and the child spontaneously says, "Mommy hits me." The child continues to talk about the pictures they look at, and the mother sits calmly on the chair. There are still a vision examination and hearing test left of the check-up. The next child on the appointment list has arrived and is sitting in the waiting room.

How much do you agree/disagree with the following statement:
I am concerned that this child has been subjected to child maltreatment, and it is necessary to further investigate this.

- Strongly disagree
- Disagree
- Partially disagree
- Neither agree nor disagree
- Partially agree
- Agree
- Strongly agree

Abbreviations: 1:PHN-public health nurse, 2:RN-registered nurse, 3:CFHC-child and family health clinic, 4:CPS-child protective service, 5:GP- general practitioner.

Describe your concerns: (This question only appears if the option "Partially agree, Agree, or Strongly agree" is selected in the question "How much do you agree/disagree with the following statement?")

Any comments: (This question only appears if the option "Strongly disagree, Disagree, Partially disagree, or Neither agree nor disagree" is selected in the question "How much do you agree/disagree with the following statement?")

How much do you agree/disagree with the following statement: I feel confident that I have the competence and knowledge to handle this case.

Strongly disagree
Disagree
Partially disagree
Neither agree nor disagree
Partially agree
Agree
Strongly agree

Any comments: (This question only appears if the option "Strongly disagree, Disagree, Partially disagree, or Neither agree nor disagree" is selected in the question "How much do you agree/disagree with the following statement?")

Which of the following actions would you implement in this situation? (Multiple selections are possible)

Examined the child naked
Evaluated the interaction between parents and child
Asked the mother about what has happened
Asked the mother about what she does when she gets frustrated
Informed the parents about my concerns
Requested permission to discuss with other agencies (daycare, etc.)
Documented and described the situation in the child's record
Offered additional home visits
Thoroughly assessed and evaluated the child's history
Scheduled a follow-up appointment
Discussed the case with colleagues internally at the CFHC
Referred to a pediatrician at the CFHC
Discussed the case anonymously with child protective services (CPS)
Referred for examination by the general practitioner (GP)
Referred for examination at a hospital/child department
Filed a report of concern with CPS
Reported to the police
None of these actions
Other actions

What other actions?: (This question only appears if the option 'Other actions' is selected in the question 'Which of the following actions would you implement in this situation?')

Case 2:
A mother and father come to the CFHC with their 18-month-old child. The family resides in Asia, but the father has been in Norway for the past two years due to a research position. The mother and child moved to the district/municipality six months ago and have been following the CFHC program since then. The family is expected to stay in Norway for 4 years. The father speaks Norwegian, while the mother speaks English. The family has had several additional consultations because the mother has expressed dissatisfaction with living in Norway since their first visit to the CFHC. The child is developing appropriately for its age. It is challenging to examine the child as it is highly skeptical of the PHN and clings to her mother. The child does not attend daycare. The mother complains to the PHN about the father that works late into the evening, and she feels very alone. The father explains that it is because he leaves work to go home two hours during the day to make lunch

Abbreviations: 1:PHN-public health nurse, 2:RN-registered nurse, 3:CFHC-child and family health clinic, 4:CPS-child protective service, 5:GP- general practitioner.

for the mother. Therefore, he must make up those hours in the evening. The mother becomes increasingly upset during the consultation. She directly addresses the father and angrily tells him in English that he is lazy and selfish. She then stands up and continues shouting in her native language at the father. The child starts crying and clings to the mother. Appears scared. The father remains silent and sits still in his chair.

How much do you agree/disagree with the following statement:
I am concerned that this child has been subjected to child maltreatment, and it is necessary to further investigate this.

- Strongly disagree
- Disagree
- Partially disagree
- Neither agree nor disagree
- Partially agree
- Agree
- Strongly agree

Describe your concerns: (This question only appears if the option "Partially agree, Agree, or Strongly agree" is selected in the question "How much do you agree/disagree with the following statement?")

Any comments:. (This question only appears if the option "Strongly disagree, Disagree, Partially disagree, or Neither agree nor disagree" is selected in the question "How much do you agree/disagree with the following statement?")

How much do you agree/disagree with the following statement: I feel confident that I have the competence and knowledge to handle this case.

- Strongly disagree
- Disagree
- Partially disagree
- Neither agree nor disagree
- Partially agree
- Agree
- Strongly agree

Any comments: (This question only appears if the option "Strongly disagree, Disagree, Partially disagree, or Neither agree nor disagree" is selected in the question "How much do you agree/disagree with the following statement?")

Which of the following actions would you implement in this situation? (Multiple selections are possible)

- Examined the child naked
- Evaluated the interaction between parents and child
- Asked the parents about what has happened
- Asked the parents about what they do when frustrated
- Informed the parents about my concerns
- Requested permission to discuss with other agencies (daycare, etc.)
- Documented and described the situation in the child's record
- Offered additional home visits
- Thoroughly assessed and evaluated the child's history
- Scheduled a follow-up appointment
- Discussed the case with colleagues internally at the CFHC
- Referred to a pediatrician at the CFHC
- Discussed the case anonymously with CPS
- Referred for examination by the GP
- Referred for examination at a hospital/child department
- Filed a report of concern with CPS
- Reported to the police

Abbreviations: 1:PHN-public health nurse, 2:RN-registered nurse, 3:CFHC-child and family health clinic, 4:CPS-child protective service, 5:GP- general practitioner.

None of these actions
Other actions

What other actions? (This question only appears if the option 'Other actions' is selected in the question 'Which of the following actions would you implement in this situation?')

Case 3:
A young couple comes for the 3-month checkup at the CFHC. The PHN has closely followed the family because the baby has had colic symptoms and been uneasy since birth. In the consultation, the parents mention that two weeks ago, they visited the emergency room. The PHN has not received any medical report and asks the parents to share what happened. The father explains that he had woken up in the middle of the night to the baby being uncomfortable and screaming. When he was preparing a bottle of milk, he discovered that the baby was bleeding from the mouth. He woke the mother up, and they went to the emergency room at 4 AM. By the time they reached the emergency room, the bleeding had stopped, but the doctors had mentioned that the frenulum under the upper lip was torn. The parents described the incident as frightening. During the examination at the CFHC, the baby shows good growth and appears well-groomed. He maintains good eye contact. During vaccination, the PHN notices a faint bruise, approximately 1.5 cm in size, on the inside of the right thigh. The baby cries inconsolably during vaccination. Both parents seem stressed by the crying and begin to shake a rattle and dance around with the baby in large movements.

How much do you agree/disagree with the following statement: 'I am concerned that this child has been subjected to child maltreatment, and it is necessary to further investigate this'.

Strongly disagree
Disagree
Partially disagree
Neither agree nor disagree
Partially agree
Agree
Strongly agree

Describe your concerns: (This question only appears if the option "Partially agree, Agree, or Strongly agree" is selected in the question "How much do you agree/disagree with the following statement?")

Any comments: (This question only appears if the option "Strongly disagree, Disagree, Partially disagree, or Neither agree nor disagree" is selected in the question "How much do you agree/disagree with the following statement?")

How much do you agree/disagree with the following statement: I feel confident that I have the competence and knowledge to handle this case.

Strongly disagree
Disagree
Partially disagree
Neither agree nor disagree
Partially agree
Agree
Strongly agree

Any comments: (This question only appears if the option "Strongly disagree, Disagree, Partially disagree, or Neither agree nor disagree" is selected in the question "How much do you agree/disagree with the following statement?")

Which of the following actions would you implement in this situation? (Multiple selections are possible)

Examined the child naked
Evaluated the interaction between parents and child
Asked the parents about what happened

Abbreviations: 1:PHN-public health nurse, 2:RN-registered nurse, 3:CFHC-child and family health clinic, 4:CPS-child protective service, 5:GP- general practitioner.

Asked the parents about what they do when frustrated
Informed the parents about my concerns
Requested permission to discuss with other agencies (daycare, etc.)
Documented and described the situation in the child's record
Offered additional home visits
Thoroughly assessed and evaluated the child's history
Scheduled a follow-up appointment
Discussed the case with colleagues internally at the CFHC
Referred to the CFHC-doctor
Discussed the case anonymously with CPS
Referred for examination by the GP
Referred for examination at a hospital/child department
Filed a report of concern with CPS
Reported to the police
None of these actions
Other actions

What other actions? (This question only appears if the option 'Other actions' is selected in the question 'Which of the following actions would you implement in this situation?')

Preventing and/or detecting child abuse during consultations at the CFHC. We remind you that the answers are anonymous, and it is important that you answer as honestly as you can. (Please answer what you do, not what you think should be done or what you believe is the correct answer.)

In my effort to prevent or detect child maltreatment:					
	every consultation	specific/targeted consultations	based on indications	never	don't know/wish to answer
I assess for risk factors/stressors in the family at					
I ask the parents about their own childhood at					
I teach/guide the parents about child maltreatment (eg. Sbs[1], persistent crying and risk factors) at					
I talk about genital mutilation at					
I have conversations about upbringing and corporal punishment at					
I assess the childs well-being and weight at					
I assess the child's physical development at					
I assess the child's social development at					

Abbreviations: 1:PHN-public health nurse, 2:RN-registered nurse, 3:CFHC-child and family health clinic, 4:CPS-child protective service, 5:GP- general practitioner.

Below, you will find a selection of tools that can be used in iscussions/educational conversations about child maltreatment (Put a check mark next to how you think they work as a starting point for conversation.)							
	Excellent	Good	Somewhat good	Somewhat poor	Poor	Very poor	Do not use
In Safe Hands (I trygge hender)							
Parent Toolkit (Foreldrepakka)							
Understanding of our body and sexuality – Empowering Toddlers (Gode råd til deg som voksen når du snakker med barn om kropp og seksualitet)							
The language assessment tool: Language4 (Språk 4)							

Do you use any other brochures/tools in discussions/education about child maltreatment?
Yes
No

Which ones? (This question only appears if the option "Yes" is selected in the question "Do you use any other brochures/tools in discussions/education about child maltreatment?")

Do you have any other suggestions for questions/resources to prevent or detect child maltreatment: (Please do not include personal information in the free text field)

Which of the following characteristics/signs in a child increase the risk of neglect, abuse, and/or sexual abuse?					
	No increased risk	Some increased risk	Increased risk	Significantly increased risk	Don’t know
Prematurity (gestational age < 36 weeks)	1	2	3	4	5
Chronic illness	1	2	3	4	5
Unkempt	1	2	3	4	5
Allergy	1	2	3	4	5
Dental Caries/ Poor oral health	1	2	3	4	5
Obesity	1	2	3	4	5
Malnutrition/ underweight	1	2	3	4	5
History of behavioral issues	1	2	3	4	5

Abbreviations: 1:PHN-public health nurse, 2:RN-registered nurse, 3:CFHC-child and family health clinic, 4:CPS-child protective service, 5:GP- general practitioner.

**Which of the following characteristics/signs in a family increase the risk of neglect, abuse, and/or sexual abuse?**					
	No increased risk	Some increased risk	Increased risk	Significantly increased risk	Don't know
Poverty	1	2	3	4	5
Social isolation					
Alcohold or drug abuse					
Both parents in full-time employment	1	2	3	4	5
Mental health issues	1	2	3	4	5
Young age of the parents.	1	2	3	4	5
Parents with history of child maltreatment from their own childhood	1	2	3	4	5

**In what way have you acquired/are you acquiring knowledge on the subject of child maltreatment?**							
	To a very large extent	To a large extent	To some extent	To a small extent	To a very small extent	To no extent	Don't know/remeber
PHN education							
The clinical guidelines for CFHC							
Courses and conferences							
Professional guidance (eg. a psychologist) at CFHC							
Literature and self-study							
Exchanging experiences with colleagues							
News, internet or media (podcasts)							

Are there any other ways you have gained/are gaining knowledge about child abuse:

Abbreviations: 1:PHN-public health nurse, 2:RN-registered nurse, 3:CFHC-child and family health clinic, 4:CPS-child protective service, 5:GP- general practitioner.

How frequently does your workplace facilitate of the following suggestions to increase knowledge related to child maltreatment?						
	<1 a year	2-5 times a year	5-8 times a year	>8 times a year	Does not offer	Don't Know
Training, courses and/or Conferences						
Exchanging experiences with colleagues						
Professional guidance (eg. a psychologist) at CFHC						
Assigned time for literature and self-study						

Experience with child maltreatment:

Have you encountered any children in your work at the CFHC whom you suspected of being victims of physical violence?

Yes

No

How often do you encounter children at CFHC where you suspect physical violence? (This question is displayed only if the option 'Yes' is selected in the question 'In your practice, have you encountered children at the health station where you have suspected that the child has been subjected to physical violence?')

<1 child a year

1-2 children a year

3-6 children a year

>6 children a year

Don't know/remember

Have you encountered any children in your work at the CFHC whom you suspected of being victims of emotional/psychological violence?

Yes

No

How often do you encounter children at CFHC where you suspect emotional/psychological violence? (This question is displayed only if the option 'Yes' is selected in the question 'In your practice, have you encountered children at the health station where you have suspected that the child has been subjected to emotional/psychological violence?')

<1 child a year

1-2 children a year

3-6 children a year

>6 children a year

Don't know/remember

Have you encountered any children in your work at the CFHC whom you suspected of being victims of neglect?

Abbreviations: 1:PHN-public health nurse, 2:RN-registered nurse, 3:CFHC-child and family health clinic, 4:CPS-child protective service, 5:GP- general practitioner.

Yes
No

How often do you encounter children at CFHC where you suspect neglect? (This question is displayed only if the option 'Yes' is selected in the question 'In your practice, have you encountered children at the health station where you have suspected that the child has been subjected to neglect?')

<1 child a year
1-2 children a year
3-6 children a year
>6 children a year
Don't know/remember

Have you encountered any children in your work at the CFHC whom you suspected of being victims of intimate partner violence?

Yes
No

How often do you encounter children at CFHC where you suspect intimate partner violence? (This question is displayed only if the option 'Yes' is selected in the question 'In your practice, have you encountered children at the health station where you have suspected that the child has been subjected to intimate partner violence?')

<1 child a year
1-2 children a year
3-6 children a year
>6 children a year
Don't know/remember

Have you encountered any children in your work at the CFHC whom you suspected of being victims of sexual abuse?

Yes
No

How often do you encounter children at CFHC where you suspect to sexual abuse? (This question is displayed only if the option 'Yes' is selected in the question 'In your practice, have you encountered children at the health station where you have suspected that the child has been subjected to sexual abuse?')

<1 child a year
1-2 children a year
3-6 children a year
>6 children a year
Don't know/remember

Have you ever contacted a GP with concerns of child maltreatment?

Yes
No

How often? (This question is displayed only if the option 'Yes' is selected in the question 'Have you ever contacted a GP due to concerns of child maltreamtment.')

<1 a year
1-2 times a year
3-6 times a year
>6 times a year
Don't know/remember

Abbreviations: 1:PHN-public health nurse, 2:RN-registered nurse, 3:CFHC-child and family health clinic, 4:CPS-child protective service, 5:GP- general practitioner.

Have you ever contacted a specialist healthcare service (e.g. pediatric department) with concerns of child maltreatment?

Yes

No

How often? (This question is displayed only if the option 'Yes' is selected in the question 'Have you ever contacted a specialist healthcare service (e.g.. pediatric department) due to concerns of child maltreatment.')

<1 a year

1-2 times a year

3-6 times a year

>6 times a year

Don’t know/remember

Have you ever reported concerns of child maltreatment to the CPS?

Yes

No

**With what type of concerns?** (This question is displayed only if the option 'Yes' is selected in the question Have you ever reported concerns of child maltreatment to the CPS?')						
	Never	1-2 times	2-5 times	6-10 times	>10 times	Don’t know/remember
Physical Violence						
Psychological/emotional violence						
Neglect						
Intimate partner violence						
Sexual abuse						

In cases where you have filed a report to CPS. How often are you called in for a collaborative meeting about the family by CPS? (This question is displayed only if the option 'Yes' is selected in the question Have you ever reported concerns of child maltreatment to the CPS?')

Never

Rarely

Almost every time

Most of the time

Always

Don't know

Any comments: (This question is displayed only if the option 'Never or Rarely' is selected in the question In cases where you have filed a report to CPS. How often are you called in for a collaborative meeting about the family by CPS?)

Have you ever reported concerns of child maltreatment to the police?

Yes

No

**With what type of concerns?** (This question is displayed only if the option 'Yes' is selected in the question Have you ever reported concerns of child maltreatment to the police?')						
	Never	1-2 times	2-5 times	6-10 times	>10 times	Don’t know/remember

Abbreviations: 1:PHN-public health nurse, 2:RN-registered nurse, 3:CFHC-child and family health clinic, 4:CPS-child protective service, 5:GP- general practitioner.

Physical Violence	1	2	3	4	5	6
Psychological/emotional violence	1	2	3	4	5	6
Neglect	1	2	3	4	5	6
Intimate partner violence	1	2	3	4	5	6
Sexual abuse	1	2	3	4	5	6

**How often are you invited for collaboration by the following entities regarding concerns for a child?**					
	>5 times/year	3-5 times/year	1-2 times/year	>1 time/year	Never
Daycare	1	2	3	4	5
GP	1	2	3	4	5
CPS	1	2	3	4	5
Police	1	2	3	4	5
Hospital/children ward	1	2	3	4	5
Child and Adolescent Psychiatric Outpatient Clinics	1	2	3	4	5

Do other professional groups contact you regarding concerns they have?

Yes

No

Who? (This question is displayed only if the option 'Yes' is selected in the question 'Do other professional groups contact you regarding concerns they have?')

**In the collaboration on families where you are concerned about a child, how much do you agree or disagree with the following statement?**							
	Strongly diagree	Disagree	Somewhat disagree	Neither disagree nor agree	Somewhat agree	Agree	Strongly agree
The collaboration between midwives and PHNs in cases of concern is good	1	2	3	4	5	6	7
The collaboration between PHNs and other professions within the CFHC in cases of concern is good	1	2	3	4	5	6	7
The collaboration between PHNs and CPS is good	1	2	3	4	5	6	7
I am invited to collaborative meetings with CPS when I have submitted a report of concern.	1	2	3	4	5	6	7

Abbreviations: 1:PHN-public health nurse, 2:RN-registered nurse, 3:CFHC-child and family health clinic, 4:CPS-child protective service, 5:GP- general practitioner.

I experience that the CPS dismiss cases of children who I am concerned.	1	2	3	4	5	6	7
I receive discharge reports from hospitals when a child has been hospitalized.	1	2	3	4	5	6	7
I perceive that CPS have a high level of expertise in children's development and needs.	1	2	3	4	5	6	7
Confidentiality limits collaboration with others	1	2	3	4	5	6	7

**How much do you agree or disagree with the following statements?**							
	Strongly diagree	Disagree	Somewhat disagree	Neither disagree nor agree	Somewhat agree	Agree	Strongly agree
To detect/prevent child maltreatment is complex and depends on many factors.	1	2	3	4	5	6	7
To detect/prevent child maltreatment requires a lot of resources and time	1	2	3	4	5	6	7
It's difficult to detect child maltreatment because we only see the children during the consultation hour.	1	2	3	4	5	6	7
It is difficult to detect child maltreatment because we see the children for such a short time.	1	2	3	4	5	6	7
It is difficult to detect child maltreatment because we have to talk about so many things.	1	2	3	4	5	6	7
If I'm concerned about a child, it constantly bug me	1	2	3	4	5	6	7
I feel alone in the responsibility when I am concerned about a child.	1	2	3	4	5	6	7
I get good support from my supervisor in difficult cases	1	2	3	4	5	6	7
It's difficult to detect child maltreatment at the CFHC because parents can cancel appointments if the child has visible bruises.	1	2	3	4	5	6	7

Abbreviations: 1:PHN-public health nurse, 2:RN-registered nurse, 3:CFHC-child and family health clinic, 4:CPS-child protective service, 5:GP- general practitioner.

It's challenging to detect child maltreatment because parents hide it from the PHN	1	2	3	4	5	6	7
It is easy to ask direct questions about child maltreatment	1	2	3	4	5	6	7
When I talk about child maltreatment, I tend to be a bit subtle	1	2	3	4	5	6	7
I'm afraid of making the situation worse for the child if I report it	1	2	3	4	5	6	7
I'm afraid of making the situation worse for the child if I report it.	1	2	3	4	5	6	7
I'm afraid of damaging my relationship with the family if I file a report.	1	2	3	4	5	6	7
I'm afraid of mistaking if I don't have obvious signs of maltreatment.	1	2	3	4	5	6	7

Comments:

Does the CFHCr where you work have an action plan for addressing child maltreatment?

Yes
No
Don't know

In cases where you suspect or uncover child maltreatment/neglect, how often do you use this action plan?
(This question is only shown if the option "Yes" is selected in the question "Does the CFHC where you work have an action plan for addressing child maltreatment against children?")

Always
Very often
Often
Sometimes
Rarely
Very rarely
Never
Don't know/Prefer not to answer

**What following measures do you believe can help in the work of preventing/detecting child maltreatment at the CFHC? (Multiple answers possible)**	
More consultations	
Home visits	
Establish first meeting with the family during pregnancy	
Available communication tools	
Specific measures described in the guidelines	
Improved collaboration with CPS	
More training	
Possible to get guidance and support	
Specific questions to ask the parents	

Abbreviations: 1:PHN-public health nurse, 2:RN-registered nurse, 3:CFHC-child and family health clinic, 4:CPS-child protective service, 5:GP- general practitioner.

More time for individual follow-up	
Clear procedures in the clinical guideline	
Other suggestions	

Your suggestions: (This question is only shown if the option "other suggestions" is selected in the question " What following measures do you believe can help in the work of preventing/detecting child maltreatment at the CFHC?"

Do you have any comments or feedback before you finish the survey?

Yes
No

Comments: (This question is only shown if the option "Yes" is selected in the question "Do you have any comments or feedback before you finish the survey?")

Thank you so much for taking the time to respond to this survey. Press send to finish.

Abbreviations: 1:PHN-public health nurse, 2:RN-registered nurse, 3:CFHC-child and family health clinic, 4:CPS-child protective service, 5:GP- general practitioner.
